# Extracellular Vesicles in Ophthalmology: From Natural Nanocarriers to Engineered Therapeutics

**DOI:** 10.3390/bioengineering13030275

**Published:** 2026-02-27

**Authors:** Christopher Flores, Fabiana Mastantuono, Lu Huang, Tina B. McKay, Grace M. Coyne, Brenna Hefley, Brenda Vasini, Dimitrios Karamichos, Menglu Yang

**Affiliations:** 1 Department of Ophthalmology, Schepens Eye Research Institute of Mass Eye and Ear, Harvard Medical School, Boston, MA 02114, USA; cf11@williams.edu (C.F.); f.mastantuono@ufl.edu (F.M.); lhuang31@meei.harvard.edu (L.H.); gmcoyne@meei.harvard.edu (G.M.C.); 2 Williams College, Williamstown, MA 01267, USA; 3 Department of Biomedical Engineering, College of Engineering, University of Florida, Gainesville, FL 32611, USA; 4 Mass General Brigham Department of Anesthesiology, Massachusetts General Hospital, Boston, MA 02114, USA; tmckay@mgh.harvard.edu; 5 North Texas Eye Research Institute, University of North Texas Health, Fort Worth, TX 76107, USA; brenna.hefley@unthsc.edu (B.H.); brenda.vasinirosell@unthsc.edu (B.V.); 6 College of Biomedical and Translational Sciences, University of North Texas Health, Fort Worth, TX 76107, USA; 7 Department of Family Medicine, Texas College of Osteopathic Medicine, University of North Texas Health, Fort Worth, TX 76107, USA

**Keywords:** extracellular vesicles, ocular diseases, drug delivery, surface modification, synthetic vesicles, mimetic vesicles, gene therapy, EV storage

## Abstract

Extracellular vesicles (EVs) are increasingly recognized as programmable bioactive carriers in non-viral gene delivery and adaptable bioengineering platforms. Beyond their roles as natural nanocarriers in intercellular communication, EVs can promote ocular surface repair and retinal neuroprotection with potential for low immunogenicity and high biocompatibility. Bioengineering now enables cargo encapsulation, surface targeting, and integration of EVs with biomaterial platforms to enhance tissue penetration, retention, and precision delivery. The emergence of induced pluripotent stem cell-derived EVs (iMSC-EVs) offers improved batch uniformity and potential for personalized therapy. However, progress hinges on resolving knowledge gaps in ocular EV biology, standardizing isolation and storage, scaling reproducible manufacturing, and executing focused clinical trials. We synthesize the current developments and outline how EVs are moving from biological mediators to engineered therapeutics to accelerate the translation of EV diagnostics and therapeutics for eye diseases.

## 1. Introduction

**Figure 1:**
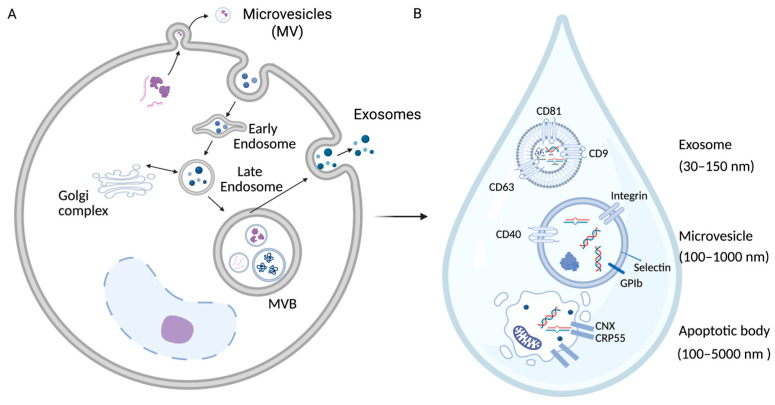
Biogenesis and cargo of tear-derived extracellular vesicles. (**A**) Biogenesis of extracellular vesicles (EVs), including exosomes, microvesicles (MV), and apoptotic bodies. (**B**) Size, cargo and markers for each type of EV.

The eye is a unique therapeutic environment that is highly accessible but is tightly protected by anatomical and immune barriers such as the tear film, corneal epithelium, and blood–retina barrier. These maintain ocular immune privilege and homeostasis but also limit the penetration and retention of conventional drugs. Extracellular vesicles (EVs), nanosized lipid bilayer vesicles secreted by nearly all cell types (Figure 1A). They naturally cross biological barriers and mediate cell–cell communication by transferring cargo that reflects their parent cells, including proteins, lipids, and nucleic acids [1,2]. EVs are broadly classified into exosomes (30–150 nm), micro vesicles (100–1000 nm), and apoptotic bodies (100–5000 nm) [3] (Figure 1B). Their intrinsic biocompatibility, low immunogenicity, and ability to be engineered for cargo loading and surface targeting make EVs promising tools for ophthalmic diagnosis and therapy [4,5]. This review integrates (i) the biological functions of EVs in ocular health and disease, (ii) preclinical evidence for therapeutic efficacy, (iii) bioengineering strategies to enhance EV function, and (iv) translational challenges and opportunities.

### Human Eye Structure and Biological Roles of EVs

**Figure 2:**
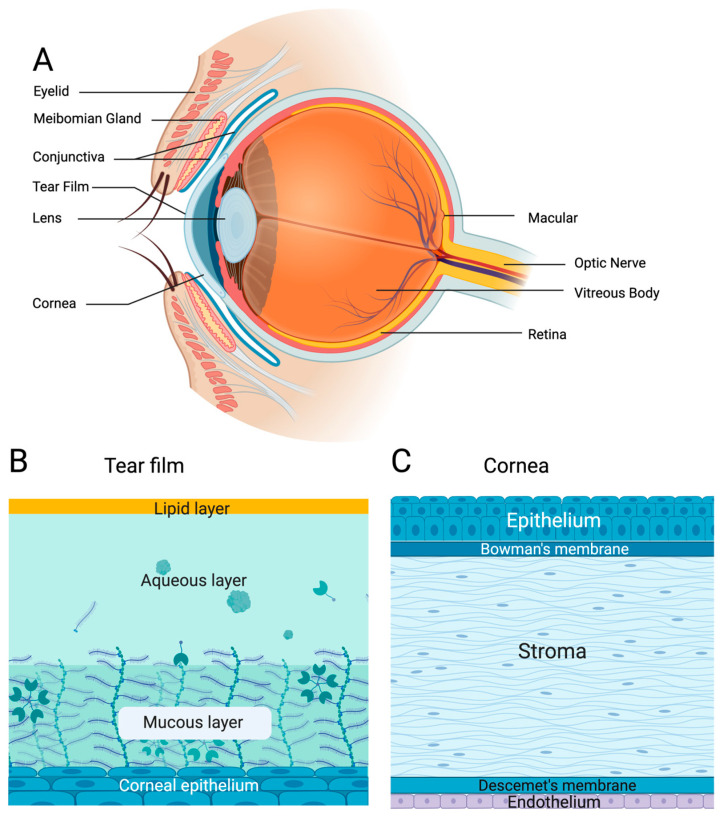
The structure of the human eye. (**A**) The external and internal anatomical structures responsible for protecting and maintaining ocular function. (**B**) The tear film is composed of lipid, aqueous, and mucous layers that stratify on top of the surface of the corneal epithelium. (**C**) The cornea is composed of 3 major cellular layers (epithelium, stroma, and endothelium) and 2 acellular basement membranes (Bowman’s and Descemet’s).

The human eye is a highly specialized sensory organ that enables vision. The visual function depends on the coordination of multiple precisely organized ocular structures that maintain optical clarity and neural connectivity (Figure 2A). On the outer surface of the eye, tear film acts as a barrier between the eye and the environment [6]. Comprising lipid nonpolar and polar aqueous layers, tear film contains a variety of electrolytes, lipids, and proteins (Figure 2B). These layers assist in maintaining eye moisture, clearing debris, and defending against pathogens from the environment, in turn reducing inflammation and promoting healing [6]. Below the tear film lies the cornea, which is a clear, five-layer structure containing the epithelium, Bowman’s membrane, lamellar stroma, Descemet’s membrane and the endothelium (Figure 2C) [7]. Corneal nerve fibers travel through the stroma and epithelia, providing essential support for those innervated structures [7]. Surrounding the cornea is the conjunctiva, which is an epithelial layer responsible for blocking pathogens and maintaining moisture on the anterior segment of the eye [8]. In the inner surface of the eye lies the retina [7], known as the ‘sensor’ of the eye, since it is where light is focused to interpret visual signals [9]. The neural retina layer is the inner layer (surrounding the vitreous cavity), and the outer layer (surrounded by the choroid and sclera) is the retinal pigment epithelium [7]. Inside the retina, photoreceptor cells (rods and cones) receive and process light signals [7]. Next, these light signals are converted to nerve impulses that travel through photoreceptors, bipolar cells and ganglion cells, which form a pathway to the optic nerve (made of axons from ganglion cells), leading directly to the brain for visual processing [7].

The eye maintains a separate and highly regulated compartment, controlled by anatomical and physiological barriers [9]. Notable examples include the blood–retina barrier, the tight junctions between retinal pigment epithelial cells, and the tight junctions of the corneal epithelium [10]. Therefore, during diseases when drug penetrations are necessary, mechanisms are needed to overcome these barriers and deliver the molecule to the targeted tissue.

EVs play multifaceted roles in maintaining ocular health and contributing to disease. They act as vital mediators of intercellular communication among epithelial, stromal, neuronal, and immune cells, carrying molecular cargo that reflects the physiological state of their parent cells [1]. Through cargo, EVs, the non-replicating nanocarriers, regulate processes such as epithelial maintenance, angiogenesis, and immune modulation [11,12]. In pathological conditions, however, these signaling mechanisms become dysregulated. Altered EV composition and secretion have been reported in age-related macular degeneration (AMD), diabetic retinopathy, and chronic ocular inflammation [4]. Because EVs circulate in all major ocular biofluids, including tears, aqueous humor, and vitreous humor, they dynamically reflect local and systemic changes, making them both indicators of ocular physiology and potential vehicles for therapeutic intervention [13]. As an example, corneal epithelial cells readily uptake EVs [14] with fluorescently labeled exosomes having been shown to penetrate the stroma and fuse with resident keratocytes [15], particularly when the epithelial barrier is compromised [16]. By leveraging the intrinsic penetrative properties of EVs, researchers have expanded their potential beyond natural cellular communication, establishing EVs as promising therapeutic tools.

## 2. Stem Cell EV Therapies

### 2.1. iPSC-Derived MSC EVs as Therapeutics

EVs possess significant therapeutic potential in both anterior and posterior ocular segments. The dominant pathways regulated by EVs in corneal epithelial cells are the formation of the ternary ribosomal complex, translation initiation complex formation, and translational elongation [17]. EVs from Mesenchymal stem cells (MSCs) have recently attracted numerous research interests. MSCs are multipotent adult stromal cells that have self-renewal capacity, multilineage differentiation, immunomodulatory effects, and trophic factor release [18]. Their therapeutic effect has been reported in multiple diseases such as Systemic Lupus Erythematosus (SLE), Multiple Sclerosis (MS), and Graft-versus-Host Disease (GVHD) [19]. In the eye, the therapeutic potential of MSCs has been explored in dry eye disease [20], ocular chemical burn [21], and glaucoma [22]. Compared to MSCs, MSC-EVs are cell-free therapeutic systems with high biocompatibility, safety, and low immunogenicity that still carry therapeutic efficacy. MSC-EVs also accelerate corneal epithelial wound healing by promoting cell proliferation and migration while suppressing inflammation and fibrosis, thereby restoring transparency through modulation of cytokine signaling and extracellular matrix remodeling [23,24]. In the retina and optic nerve, MSC-derived and induced pluripotent stem cell-derived EVs (iMSC-EVs) protect photoreceptors and retinal ganglion cells, enhance axonal regeneration, and attenuate apoptosis through activation of PI3K/AKT signaling and inhibition of NF-κB-driven inflammation [25]. The immunomodulatory effects of EVs further help to maintain ocular immune privilege by balancing microglial activation and T-cell responses [23]. Multiple murine and rat models of ocular diseases and injury such as glaucoma, Sjögren’s disease, optic nerve crush (ONC), retinal ischemia, Benzalkonium chloride (BAK) induced keratopathy, etc., treated with bone marrow-derived MSC EVs (BM-MSC-EVs) revealed a reduction in neuroinflammation, cell proliferation, promotion of cell survival (RGCs, epithelial cells, etc.), and even functional improvement in ischemia and glaucoma models [26]. Whether BM-MSC-EVs were administered by intravitreal injection in the lacrimal glands or eye or topically or systematically administered, decreased expression of inflammatory markers such as TNF-α, IL-1β, IL-6, IFN-γ, etc., was observed across models [26]. MSCs exhibit anti-apoptotic, anti-inflammatory, neuroprotective effects and nerve regeneration in glaucoma, optic nerve injury, and diabetic retinopathy [27]. While BM-MSCs are among the most studied MSC types, variability with age and functionality, possibly limiting therapeutic effects, as well as painful and invasive harvesting procedures, highlight BM-MSC limitations [26]. Umbilical cord-derived MSCs and Adipose-derived MSCs require noninvasive or minimally invasive procedures, respectively; however, differences in processing bring forth concerns in clinical applications, and there is limited clinical data compared to other MSC types, such as BM-MSCs [26].

Despite the improvement to MSCs, MSC-EVs still have inherent limitations, such as variation in biological properties due to discrepancies in the donor, tissue source, and culture conditions, as well as limited expandability. Induced pluripotent stem cells (iPSCs) offer theoretically unlimited expandability and increased uniformity through clonal reprogramming, but their direct therapeutic use is limited by concerns related to residual pluripotency. Therefore, iMSC-EVs have garnered attention as next-generation, cell-free therapeutics due to their superior uniformity, scalability, and prolonged bioactivity, like their immunosuppressive efficacy, compared to conventional MSC-EVs, which have variation in biological properties due to discrepancies in the donor, tissue source, and culture conditions, as well as limited expandability [28,29]. A detailed comparison between MSC, iPSC, MSC-EV, and iMSC-EV is listed in Table 1. Early passage iMSC-EVs demonstrate stronger immunomodulatory potency than late-passage EVs in TLR4-stimulated splenocytes and in a mouse model of primary Sjögren’s syndrome [30]. In a rat optic nerve crush model, iMSC-EVs improved retinal ganglion cell survival, reduced axonal atrophy, and enhanced regeneration via PI3K/AKT signaling [25]. Similarly, iMSC-EVs carrying the Splicing factor proline/glutamine-rich (SFPQ) upregulated HDAC1 and protected Müller cells from hypoxia-induced injury through suppression of HIF-2α [31]. When it comes to the regulation of the immune response, iMSC-EVs showed effective immunomodulation by inhibiting T-cell proliferation and inducing macrophage polarization as effectively as their parent cells. They also induced regeneration in in vitro scratch assays as effectively as hUCMSC-EVs [32]. Another application is using iMSC-EVs combined with electroacupuncture to exert neuroprotective effects by regulating the IL-33/ST2 axis and inhibiting microglia and astrocyte formation [33]. The limited number of ophthalmology-specific iMSC-EV studies highlights a current gap in the field, motivating the inclusion of insights for systemic disease models essential for informing future ocular research. These studies support that iMSC-EVs are beneficial for immunomodulation, regeneration, and anti-inflammation, which can be crucial for targeting a variety of ocular diseases and injuries (Table 2).

Another advantage for using iMSC-EVs is the ability to develop from a patient’s own cells. This approach elicits minimal immune responses and exhibits prolonged circulation, representing an ideal platform for personalized medicine [34,35]. For instance, iMSC RAB22A-induced EVs containing activated STING have been shown to trigger IFN-β expression and antitumor immunity in mice [36]. EVs can also be engineered to deliver therapeutic agents, with dosing-optimized testing in patient-specific iPSC-derived organoids to minimize the systemic toxicity of a chemotherapy agent [34].

Collectively, these findings highlight the expanding role of stem cell-derived EVs, from natural nanocarriers to engineered, cell-free therapeutics that can modulate inflammation, promote regeneration, and restore tissue homeostasis in ocular diseases, while recapitulating many of the benefits of stem cell therapy without the risks associated with cell transplantation.

**Table 2:** Reported therapeutic effects of iMSC-EVs in systemic and ocular diseases.

Category	Target Application	Reported Effect	Study Model/Subject	Ref.
Regeneration and anti-inflammation	Ocular	Delayed retinal degeneration	rd10 mouse model and retinal cell co-culture	[37]
		Blood–retina barrier preservation	Mouse model and human induced iPSC culture	[38]
		Diabetic retinopathy	Rat model and cell-based assays	[39]
	Non-ocular	Ovarian repair	Mouse model and granulosa cell culture	[40]
		Diabetic wound healing	Diabetic mouse wound model and cell-based assays	[41]
		Acute kidney injury	BALB/c mouse model and HK-2/THP-1 cell lines	[42]
Immuno-modulation	Ocular	Sjogren’s improvement	Mouse model and splenocyte culture	[30]
			NOD.B10.H2b mouse model and splenocyte analysis	[43]
		Autoimmune uveoretinitis improvement	Mouse model and splenocyte culture	[44]
	Non-Ocular	Septic lung injury	Rat model and alveolar macrophage culture	[45]
		Immune regulation	Human immune cell co-culture	[30]
			iMSCs from human urinary tubular epithelial cells	[46]
Neuroprotection	Ocular	Optic nerve repair	Rat optic nerve crush model	[25]
		Retinal ganglion cell protection	Rat optic nerve crush model	[47]
		Retinal Muller cell hypoxia protection	Rat retinal ischemia/reperfusion model and Müller cell culture	[29]
	Non-Ocular	Ischemic Stroke recovery	Mouse MCAO ischemic stroke model	[16]

### 2.2. MSC-EVs in Clinical Trials

Building on preclinical findings, clinical studies have begun to evaluate the therapeutic potential of EVs due to their natural biocompatibility, molecular stability, and targeted delivery capacity (Table 3). MSC-EVs have been the most extensively investigated, demonstrating efficacy in reducing inflammation and promoting regeneration. In a phase II trial for COVID-19-related acute respiratory distress syndrome (*n* = 102), intravenous BM-MSC-EV administration reduced 60-day all-cause mortality from 47.1% to 29.4% [48]. In ocular applications, MSC-EVs improved epithelial barrier integrity and tear retention in patients with graft-versus-host disease-associated dry eye (*n* = 28) through EV-bound miR-204 [49], while intravitreal BM-MSC-EV injections in retinitis pigmentosa (*n* = 10) were well tolerated and led to moderate gains in best-corrected visual acuity [50]. Autologous platelet- and EV-rich plasma therapies have also shown reduced inflammation and accelerated wound healing in small-scale trials [51,52].

Overall, early clinical findings confirm the safety and therapeutic promise of EV-based treatments, particularly autologous EVs. However, the absence of standardized manufacturing, quality control, and large-scale production protocols remains a major barrier to regulatory approval. Continued optimization of isolation, characterization, and dosing strategies will be essential to advance EVs from experimental therapeutics toward Food and Drug Administration (FDA)-approved clinical applications. Despite the limited understanding of iMSC-derived EVs in ocular disease, encouraging evidence from studies utilizing MSC-derived EVs provides a foundation for future ocular applications. In one study, tonsil MSC-derived EVs had preventative and therapeutic effects for retinal degenerative disease, partially because of their ability to regulate intracellular oxidative stress [53]. In another study, EVs from umbilical cord-derived MSCs significantly reduced retinal damage, increased the number of retinal ganglion cells, and inhibited the activation of caspase-3 and cell apoptosis, which helped alleviate optic nerve injury caused by chronic ocular hypertension [54].

**Table 3:** Select clinical studies evaluating cell-derived EVs as therapeutics.

EV Source	Study Design	Population	No. of Subjects Enrolled	Route Administered	Primary Outcome	Results	Ref.
BM-MSC	Double-anonymized randomized placebo-controlled Phase 2 trial	Patients with moderate to severe ARDS with COVID-19	102	IV	All-cause mortality at 60 days	Reduced 60-day mortality from 47.1% placebo to 29.4% EV-treatment	[48]
	Phase I prospective study, open-label, single-center study	Patients with retinitis pigmentosa with a best-corrected visual acuity of 20/60 to 20/400	7	Intravitreal	BCVA improved in some after 1 to 3 months post-injection, but worsened after 6 months to two years post-injection due to disease progression	Significant improvement in vision based on NEI Visual Function Questionnaire	[55]
Human Wharton’s Jelly MSCs (UC-MSCs)	Triple-blinded, randomized controlled Phase I/II clinical trial	Patients with primary Sjögren’s disease with associated dry eye syndrome symptoms	8	Topical eye drops	OSDI decreased and normal corneal fluorescein staining increased in treated group	Significant improvement in tear secretion, OSDI score, corneal fluorescein staining, and TFBUT	[56]
Autologous malignant ascites	Randomized phase I clinical trial	Patients with stage III or IV colorectal cancer	54	Subcutaneous	Safety and induction of tumor-specific anti-tumor immunity and CTL response	Positive induction of anti-tumor immunity and CTL response	[57]
Autologous platelet and EV-rich plasma	Prospective randomized controlled clinical trial	Patients with CPTBCI	25	Auricular	CPTBCI foci area and quality of life assessments	Improvement in CPTBCI and quality of life measurements	[51]
MSC	Prospective clinical trial	Patients with refractor GVHD-dry eye disease	14 (28 eyes)	Ophthalmic	OSDI score, tear film breakup time, corneal fluorescein score	Lower OSDI, reduced fluorescein score, higher tear film breakup time	[49]
Autologous BM-MSC	Non-randomized phase I clinical trial	Patients with severe retinitis pigmentosa	14	Intravitreal	Safety profile, BCVA, VF, CST	Improvement in BCVA	[50]

(Abbreviations: ARDS, acute respiratory distress syndrome; BM-MSC, bone marrow-derived mesenchymal stem cell; BCVA, best-corrected visual acuity; TFBUT, tear film breakup time; UC-MSC, umbilical cord-derived mesenchymal stem cell; CPTBCI, chronic postoperative temporal bone cavity inflammation; CST, central subfield thickness; GVHD, graft-versus-host disease; IV, intravenous; OSDI, ocular surface disease index; visual field (VF)).

### 2.3. Challenges in MSC-EV Therapy Development

Although natural, unmodified EVs have shown benefits in anti-fibrosis [16], regeneration [58], anti-inflammation [59], and act as a source of biomarkers [3] for multiple eye diseases, several limitations exist that limit their clinical application. The major limitation is that natural EV isolates remain highly heterogeneous [60,61]. This issue has drawn the attention of the International Society for EVs, which has established the MISEV guidelines to standardize reporting and characterization [62]. This heterogeneity contributes to an unpredictable immunogenic profile, as EVs are not immunologically inert and can provoke inflammatory responses, depending on the source [63,64]. Novel technologies like the ultrafast EXODUS isolation system [65] have been developed to address the underlying challenge of inconsistent preparations [66,67]; however, heterogeneity originating from the parent cells remains a persistent issue. Even though iMSCs can be beneficial in providing a growth advantage compared to MSCs, their resulting EVs still face the challenge of functional variability. Thus, it is still important to characterize functionality batch-to-batch for downstream applications [68]. Additionally, proteomic analysis indicated that EVs derived from MSCs have a more robust profile of proteins with higher expression levels than those derived from iMSCs [28]. This discrepancy underscores the critical role of bioengineering technologies in enabling precise control over EV components and enhancing consistency. In the following sections, we will provide a detailed overview of the current technologies and strategies employed in EV bioengineering.

## 3. Bioengineering Strategies to Enhance EV Therapeutics

To address the challenges of natural EVs in their heterogeneity, short tissue retention, and limited targeting specificity, which restrict their clinical application [3,16,58,59], diverse bioengineering strategies have been developed to enhance EV stability, precision, and therapeutic efficacy. EV bioengineering is an advancing discipline aimed at overcoming the inherent heterogeneity and limited therapeutic efficiency of naturally derived EVs. It is a process to tailor EVs to achieve enhanced targeting specificity, controlled cargo delivery, and improved production consistency through the application of bioengineering strategies such as genetic modification of donor cells, surface functionalization, and cargo loading techniques. The primary goal of EV bioengineering is to generate standardized, scalable, and functionalized vesicles suitable for applications in clinics.

### 3.1. Top Down: Surface Modification Strategies

**Figure 3:**
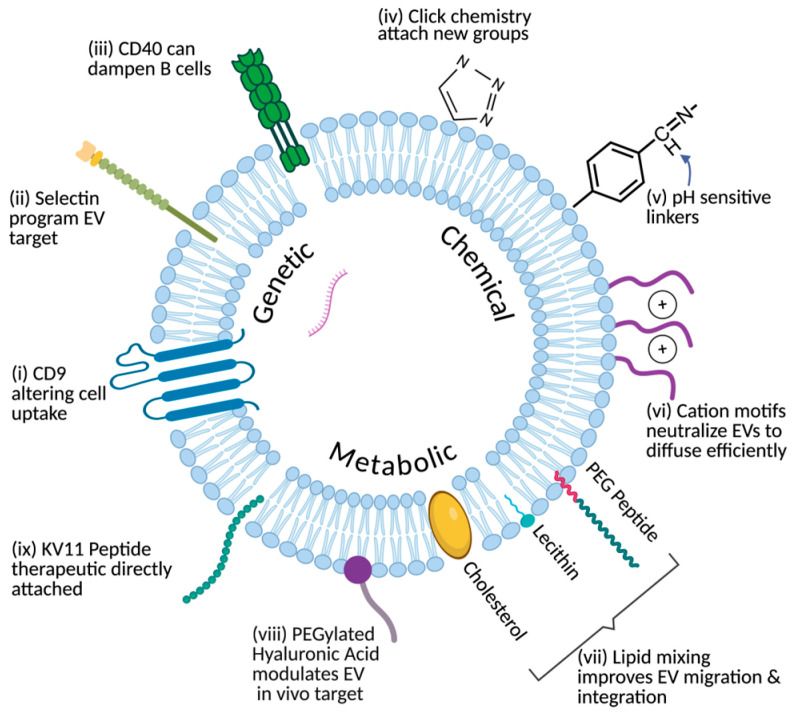
Schematics of the top-down approach to EV bioengineering. (**i**–**iii**) Genetic: Express specific proteins or ligands on EV surface during biogenesis; (**iv**–**vi**) Chemical: Add chemical structures to isolated EVs for targeting or responses; (**vii**–**ix**) Metabolic: Alter surface makeup, install “handles” on surfaces of parent cells for later use.

Top-down approaches enable precise addition of targeting ligands or functional molecules to EV surfaces. These methods modify the parent cell or the vesicle membrane directly, preserving the EV’s native biological identity while enhancing functionality. Common top-down approaches include genetic engineering, chemical modification, and metabolic labeling (Figure 3).

Genetic engineering modifies parent cells to express fusion proteins that are selectively incorporated into EV membranes during biogenesis. Target peptides or functional domains can be genetically fused to EV-enriched transmembrane or sorting proteins, including Lamp2b, CD9, CD63, and related scaffolds, enabling stable surface display on secreted vesicles [69]. For example, human umbilical cord mesenchymal stem cells engineered to express RVG-Lamp2b and Netrin-3 produced EVs that preferentially targeted neural tissue and enhanced Schwann cell-mediated repair in peripheral nerve injury models [70]. Similarly, bone marrow mesenchymal stem cell-derived EVs coexpressing RVG-Lamp2b and neurotrophin-3 promoted neuronal targeting, reduced neuroinflammation, and supported myelin repair in a facial nerve injury model [71]. In another study, EVs were modified to overexpress different transmembrane scaffolds, such as with CD63-GFP scaffolds, which were found to decrease expression of CD81 on the membrane surface and alter cell-uptake functions, with engineered EVs taken up by recipient cells at a higher rate [72]. Another group successfully engineered EVs expressing P-selectin binding peptide whose treatment in AKI mice dampened inflammatory cell infiltration, pathological damage, and fibrosis [73]. A last example is engineered EVs presenting CD40, which intercepted CD40/CD40L interactions between B cells and CD4+ T-cells, leading to B cells weakening [74]. In addition to this, when drug-loaded, these EVs relieved autoimmunity by reducing autoantibodies, proinflammatory cytokines, and immune cells [74]. In choroidal neovascularization models of mice and non-human primates, exosomes from regulatory T-cells conjugated with anti-VEGF antibodies specifically targeted neovascularization sites and effectively suppressed neovascularization [27]. Although these cases were outside the eye’s periphery, they highlight the potential for ophthalmology to implement similar genetic strategies for greater ocular targeting.

Chemical modification techniques are alternative strategies that often rely on click chemistry, which enables synthetically simple joining of molecules. This method allows attachment of peptides, antibodies, or imaging agents without compromising membrane integrity [75]. For example, azido groups were introduced onto EV surfaces, enabling click chemistry conjugation of toll-like receptor 9 agonists that enhanced dendritic cell activation and achieved strong antitumor immunity and tumor-free survival in lymphoma and melanoma models [76]. In another study, azide handles were introduced onto small EVs and conjugated to an ASGR1-specific single-chain antibody fragment using click chemistry, generating hepatocyte-targeted EVs that improved liver function in acute liver failure models [77]. Another chemical technique involves attaching pH-sensitive linkers to the surface, which in one case provided easier conjugation of antibodies to EVs [78]. Additionally, as topical treatment of vitreoretinal diseases remains challenging due to slow corneal uptake, neutralizing the charge of the EV surface, without damaging morphology or size, via cationic motifs resulted in two-fold faster steady state diffusivity through bovine corneas to deliver mRNA to retinal photoreceptors [79]. In addition, to improve choroidal neovascularization targeting, exosomes have been bioengineered with an ASL membrane Anchor (BODIPY), a Spacer (PEG), and targeting Ligands (cyclic RGD peptide) that showed improved retinal penetration without inducing gliosis [80]. These last examples represent progress in applying techniques from other fields to ocular bioengineering.

Another technique, metabolic labeling, introduces modified molecular precursors, such as azide-labeled sugars, to parent cells so that reactive groups become incorporated into the glycoproteins and lipids of the resulting EVs. In an earlier example, EVs were metabolically tagged with azido-sugars to construct click-ready EV vaccines, significantly enhancing antigen presentation and T-cell activation [76]. These “chemical handles” have been used for conjugation, imaging, or selective enrichment as well [81]. Researchers have achieved in vivo metabolic glycan labeling by administering azido-sugars to tumor-bearing mice, enabling capture of nascent EVs on a click-enabled microfluidic chip and revealing secretion dynamics during anti-PD-L1 therapy [82]. Additionally, metabolically glycoengineered cells with PEGylated hyaluronic acid handles demonstrated the method as a safe approach for surface modification of EVs to modulate in vivo fate [81]. Lipid mixing technology is another metabolic technique, which, through engineering peptides onto the surface of MSC-derived EVs that were then loaded with anakinra, an antagonist of the IL-1 receptor, promotes targeted repair of retinal degeneration in mice [83]. Lastly, another example is direct linkage, as seen with the anti-angiogenic peptide, KV11, onto the EV surface to inhibit pathological retinal angiogenesis [84]. As mentioned, topical delivery remains a challenge, and this approach displays progress towards addressing that.

Overall, top-down surface modification enables precise and versatile tuning of EV surfaces through genetic, chemical, or metabolic strategies, enhancing their targeting specificity and translational potential while preserving their natural biocompatibility.

### 3.2. Bottom Up: Synthetic and Mimetic Vesicles

**Figure 4:**
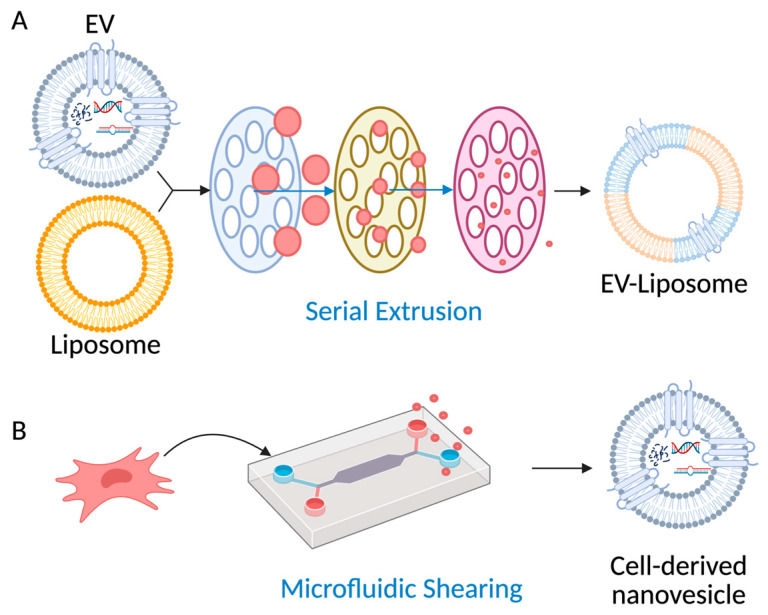
Schematics of the bottom-up approach to EV bioengineering. (**A**) Hybrid EV–liposome generated by serial extrusion; (**B**) Cell-derived nanovesicles (EV-mimetic) synthesized via microfluidic shearing. EV, extracellular vesicle.

Bottom-up strategies offer a path to overcome biological variability and scalability limitations, illustrated in (Figure 4). EV mimetics generated through extrusion or microfluidic shearing achieve yields 10–100 times higher than natural EVs while retaining select membrane proteins and biological functionality [85,86,87]. A recent review highlights that engineering EVs for ocular delivery is an area of intense interest, though it is still fraught with challenges [5]. Hybrid EV–liposome systems offer controllable composition and scalable liposome manufacturing while retaining a subset of native targeting ligands [88]. For example, nanovesicles produced by serial extrusion of MSCs showed comparable cargo profiles to naturally secreted EVs but achieved significantly higher yields and enhanced cardiac repair in a myocardial infarction mode [89]. Similarly, cell-derived nanovesicles generated through microfluidic shear disruption exhibited over a 15-fold increase in yield relative to natural EVs while maintaining key size distribution and surface marker expression [90].

Beyond yield optimization, recent studies have focused on improving the immune stealth of EV-based systems to prolong circulation and reduce clearance through hybridization. Such is the case with EVs that are being bioengineered to be “stealthier” through stable or transient overexpression of CD47, enriching vesicle surfaces with “don’t eat me” signals [88]. For instance, hybrid EV–liposome vesicles that combine synthetic membrane stability with native surface proteins were developed, resulting in reduced immune recognition and extended systemic circulation [85]. Similarly, extracellular vesicles expressing signal regulatory protein α (SIRPα) were engineered to block CD47-mediated immune evasion, achieving tumor-specific T-cell activation and potent antitumor effects with reduced hematologic toxicity in vivo [91]. These studies illustrate how bottom-up vesicle design, from high-yield synthetic mimetics to immune–evasive hybrids, enables scalable, customizable, and potentially more clinically adaptable alternatives to natural EVs (Table 4).

## 4. EV as Delivery Tools

### 4.1. Cargo Loading and Genetic Modulation

EVs can be loaded with therapeutic molecules (e.g., drugs, proteins, or nucleic acids) to enhance or tailor their effects for diagnostic and therapeutic applications, contributing to their diverse, biologically active properties. Anti-inflammatory compounds such as anakinra or small RNAs targeting fibrotic and angiogenic genes have been encapsulated for localized delivery to inflamed ocular tissues. Engineered EVs have successfully transported mRNA, siRNA, and CRISPR/Cas9 complexes for gene modulation with minimal immune activation. For example, EVs carrying therapeutic microRNAs inhibited corneal neovascularization in corneal injury models [92]. Functionally, EV cargo includes transfer RNA (tRNA) fragments and ligases, such as alanine-tRNA, asparagine-tRNA, and aspartate-tRNA, that regulate translation and inflammation in physiological and pathological contexts [17,93,94,95,96,97,98,99].

Gene therapy is a biomedical approach that introduces functional genetic material into cells to correct or replace defective genes. It requires a delivery system that is precise and efficient enough to ensure targeted uptake and sustained gene expression, as well as a minimal immune response. However, the current most popular gene delivery using viral vectors suffers from limitations such as induced immunogenicity [100], restricted gene-carrying capacity [101] and risks of insertional mutagenesis [102]. Engineered EVs are a developing delivery tool for targeted gene therapy. EVs can penetrate biological barriers, improve biofluid stability and circulation, and have a decreased probability of inducing adverse reactions compared to viral vectors, non-viral transfection, and synthetic nanocarriers [61,103]. CRISPR/Cas9 is a powerful gene editing system allowing for the insertion of new genetic material into specific areas of the genome [100]. However, the Cas9 protein (~160 kDa) struggles to cross cell membranes due to its large size, and current viral or non-viral delivery methods, such as adenoviral vectors, often trigger immune responses [100,101]. Small EVs (sEVs) can effectively deliver CRISPR/Cas9 systems to achieve precise gene editing [102]. Further research is needed to optimize the use of EVs for CRISPR/Cas9 delivery, as well as to improve isolation and purification methods for ocular EVs [60]. Nevertheless, CRISPR/Cas9-loaded EVs hold great promise for treating retinal degeneration, corneal injury, and dry eye disease, with several therapeutic approaches already under development [60]. For plasmid DNA (pDNA), besides loading using electroporation or through hybrid particles with preloaded liposomes, human megakaryocyte-derived EVs (huMkEVs) were newly introduced to deliver pDNA, enhancing hematopoietic recovery in mice in NOD-scid IL2Rγnull mice [104]. In ophthalmology, these approaches could enable targeted genetic modulation in retinal degeneration, corneal injury, and dry eye disease [60].

In addition, cell therapy can also benefit from the EV delivery system. In 2021, FOXF1 was used to reprogram nucleus pulposus (NP) cells from autopsy and surgery to anticatabolic and anti-inflammatory states, the opposite of their state when intervertebral disk (IVD) degeneration is present [103]. Transcription factors were successfully delivered in human NP cells and mouse IVD cells using EVs with limited cytotoxicity and upregulation of genes of interest, and hallmarks of healthy IVD were observed, such as GAG accumulation and decreased expression of inflammatory cytokines [103]. Therapy for posterior capsule opacification (PCO) has been developed using intraocular lenses modified with lens epithelial cell EVs to slow the progression of vision loss in rabbit models [60]. These results provide proof-of-concept for using EVs as specific genetic modification tools in cell therapy.

### 4.2. Biomaterial-Based Delivery Platforms

Biomaterial-based delivery platforms have been used in ophthalmology to deliver small molecules, proteins, and cells, but these approaches often result in burst release, limited bioactivity duration, or poor tissue retention. EVs incorporated into biomaterial platforms have emerged as a strategy to preserve their complex, multi-cargo bioactivity while avoiding challenges associated with cell-based therapies and repeated drug dosing. Given the inherent tendency of EVs to degrade rapidly and be cleared from target tissues, an increasing number of studies have explored incorporating EVs into biomaterials such as hydrogels, lacrimal plugs, contact lenses, or nanofiber scaffolds, which prolongs release, enhances cargo stability and improves ocular retention while maintaining EVs’ native intercellular signaling functions [105,106,107]. In one study, MSC-EVs were loaded into a 3D-bioprinted scaffold with an alginate core and sheath made from carboxymethyl cellulose and alginate lyase. It provided sustained EV release for chronic wound management and biodegradability [106]. Although research on utilizing scaffolds for ocular applications remains limited, one group developed a Polylactic-co-glycolic acid (PLGA) electrospinning nanofibrous scaffold (PLGAENS), loaded with MSC-EVs, which significantly improved cornea and retinal healing [107]. These findings have driven increasing interest in delivery systems that offer both structural support and fine control over EV release kinetics.

Among diverse biomaterial scaffolds explored for EV delivery, hydrogels are particularly attractive due to their high-water content, biocompatibility and porous three-dimensional networks that enable EV encapsulation and sustained release [108]. Their stiffness, degradation state, and mesh size can be tuned through polymer selection and crosslinking strategies, allowing hydrogels to be adapted to specific therapeutic and anatomical requirements [96]. Sodium alginate hydrogels are commonly used for 3D scaffold bioprinting; however, they often exhibit poor mechanical properties and rapid release kinetics. To address these limitations, a sodium alginate–silk fibroin printed hydrogel was developed, which demonstrated an increased compressive elastic modulus and slower EV release [109]. This highlights how hydrogel composition can be tailored to optimize both mechanical strength and release behavior. Hydrogel components can also be engineered to enhance therapeutic benefits for different applications, including those relevant to ocular delivery. In one study, a GelMA-dopamine-EV hydrogel accelerated wound healing and promoted skin structure normalization by improving homeostasis in the healing microenvironment of diabetic wounds [98]. In another, a hydrogel wound dressing incorporating royal jelly-derived EVs and methacrylic anhydride-modified sericin achieved gradual EV release and accelerated wound healing, with a healing rate of 96.8%, by promoting cell proliferation, angiogenesis, and inflammation modulation [110].

Hydrogels offer multiple approaches for incorporating either engineered or unmodified EVs, carrying natural cargo or therapeutic agents, for ocular delivery (Table 5). One study designed thermosensitive hydrogel eye drops for ocular inflammatory disease, which improved bioavailability and provided anti-inflammatory efficacy [111]. Another developed a methacrylate-modified silk fibroin hydrogel with embedded self-assembled indocyanine green fluorescence tracer nanoparticles, creating an injectable and degradable lacrimal plug that could be tracked long-term for dry eye treatment [112]. In addition, hydrogel contact lenses with high optical transparency and biocompatibility were engineered with microchambers for rapid, noninvasive detection of tear exosomes, supporting cancer pre-screening and diagnosis [113]. Future work integrating iMSC-EVs into these established ocular hydrogel platforms could further enhance therapeutic durability and precision at the ocular surface.

Lessons from oncology, cardiology, and general surgery highlight how combining EVs with biomaterials and loading them with specific therapeutic cargo, such as stealth coatings with CD47 and membrane cloaks, hybrid liposomal systems, and smart biomaterials, can optimize potency and reproducibility [114,115,116]. Ophthalmology, having established a strong foundation with natural EV research [70] is now well-positioned to adopt these engineering strategies for next-generation ocular therapeutics [58,59]. The largest drawback of EVs is the absence of mass and stable production resulting from their lack of a standard for characterization, which hinders their clinical usage [60,61]. EVs have much potential as a delivery tool that must be standardized to expand their clinical uses. Together, these strategies offer precise, versatile control over EV biodistribution and bioactivity, significantly improving their therapeutic potential in the eye.

**Table 4:** Spectrum of bioengineered extracellular vesicles.

EV Category	Description	Advantages	Limitations	Refs.
**Natural EVs**	Exosomes, microvesicles, and apoptotic bodies naturally secreted by cells, containing proteins, lipids, RNA from the parent cell.	Inherent biological targeting and complexity. Natural compatibility with recipient cells.	Low yields. Heterogeneity between batches. Limited scalability.	[14,66,67]
**Bioengineered EVs**	Natural EVs modified via genetic, chemical, or metabolic engineering to enhance targeting, cargo loading, or immune evasion.	Combines natural targeting with enhanced specificity and function. Have potential for reduced immunogenicity.	Added complexity in production. Plus, there are regulatory hurdles.	[69,72,81,85,117]
**EV-Mimetics**	Vesicles generated from cells via mechanical extrusion, microfluidic shearing, or other top-down methods.	Higher yields (10–100 times more than natural EVs). Retain some native membrane proteins.	Still heterogeneous and possible contamination with cytosolic components.	[86]
**Hybrid EV–Liposome Systems**	Vesicles combining components of natural EVs with synthetic liposomes.	Controlled composition. Scalable production. Retains some natural targeting ligands.	Requires optimization to preserve functional proteins. Stability concerns.	[85]
**Liposomes**	Fully synthetic lipid bilayer vesicles, often loaded with drugs or nucleic acids.	High control over size, composition, and cargo. Scalable manufacturing.	Lack natural targeting and communication signals.	[118]

**Table 5:** Hydrogel-based delivery systems for extracellular vesicles (EVs) in wound healing and ocular applications.

Application Area	Hydrogel Strategy/Type	Reported Effect	Ref.
General wound healing	GelMA–dopamine hydrogel with MSC-EVs	Accelerated diabetic wound healing; promoted skin structure normalization	[119]
	Sodium alginate–silk fibroin printed hydrogel	Increased compressive modulus; slowed EV release	[109]
	Royal jelly-derived EVs with methacrylic anhydride-modified sericin	Gradual EV release; improved proliferation, angiogenesis, and wound closure (96.8% healing rate)	[110]
Ocular applications	Multifunctional hydrogel eye drops with EVs	Synergistic treatment of ocular inflammation; improved therapeutic outcomes	[111]
	In situ forming hydrogel lacrimal plug	Degradable plug for dry eye treatment; controlled EV release	[112]
	Contact lens with hydrogel microchambers	Non-invasive detection of tear exosomes; potential diagnostic application	[113]

(Abbreviations: EVs, extracellular vesicles; MSCs, mesenchymal stem/stromal cells; GelMA, gelatin methacryloyl.)

## 5. Manufacturing and Storage: Ensuring Quality and Stability

EV stability during storage presents another major challenge in translating EV-based therapies. Vesicles are highly sensitive to environmental stressors and are prone to aggregation, membrane rupture, and cargo degradation, particularly during repeated freeze–thaw cycles. Storage buffer composition strongly influences stability: phosphate-buffered saline (PBS) alone often causes aggregation and pH drift, while PBS- or HEPES-buffered saline supplemented with trehalose and human or bovine serum albumin has been shown to minimize particle loss and preserve bioactivity by preventing surface adhesion and maintaining pH stability [120,121]. Adding 10% dimethyl sulfoxide (DMSO), a cryoprotectant, can further limit degradation of EV RNA, and non-ionic surfactants such as Tween 20 have been reported to reduce aggregation and preserve RNA integrity during cryopreservation [120,122]. Trehalose addition can further prevent aggregation and increase stability. Regarding the storage container, polypropylene tubes are preferred over glass containers, which can facilitate higher EV recovery [121].

Temperature and freeze–thaw conditions are additional determinants of EV quality, and results in the literature remain inconsistent due to variations in vesicle type and biofluid of origin [122]. Exosome release and uptake tend to increase under mildly acidic conditions, while pH drift of up to one unit can occur in common buffers as temperature drops to 0 °C [122,123]. Prolonged storage at −80 °C leads to gradual declines in EV concentration and protein purity, while multiple freeze–thaw cycles cause irreversible membrane disruption. Even modest temperature variations affect morphology [124]. Storage at 4 °C typically increases mean vesicle diameter by about 10%, whereas storage at −80 °C can cause a 25% increase compared to freshly isolated samples [122]. Proteomic analyses confirm that these membranes rupture after being frozen, and that approximately 30 proteins were detected in the supernatant instead of EVs after being stored at −80 °C [125]. While serum-derived EVs can remain stable for 1 year at −80 °C, plasma EVs degrade more rapidly in concentration and purity after only 10 to 12 days at −80 °C [122]. In ocular fluids, such as tears, fresh samples contain the purest and most abundant EVs; if freezing is required, short-term freezing and minimal freeze–thaw cycles are strongly recommended to minimize EV degradation and membrane disruption, maintaining sample integrity [120,122].

Among preservation methods, cryopreservation with antifreeze agents such as DMSO, ethylene glycol, or trehalose are widely used, but must be optimized to prevent ice-crystal damage [77,112,122]. Freeze-drying not only offers room-temperature storage with easy recovery via water addition but also reduces damage to biological samples as it is dehydrated and dried under low temperature and vacuum conditions, which is especially useful for temperature-sensitive samples [77,122,126]. Spray-drying is optimal for heat-sensitive constituents and provides a cost-effective option requiring further validation, because procedures like atomization of EV solutions, and the optimal rate of EV solution feed, atomization pressure, and temperature can damage EVs [122,126]. In practice, combining optimized cryoprotective buffers with non-crystallizing excipients and low-adsorption containers currently provides the most effective strategy to maintain EV stability and reproducibility across studies. Optimized cryopreservation or lyophilization protocols, ideally validated for each EV subtype, are critical for long-term storage.

## 6. Future Perspectives and Challenges

The clinical translation of EV applications in ophthalmology will depend on the standardization of EV isolation, dosage, storage, and bioengineering strategies. Sufficient EV purity and yield remain bottlenecks [49], and even when adequate quantities of EVs are isolated, uncertainties remain around optimal EV storage conditions [110,127]. In addition, limited understandings of the effects of EV heterogeneity on cells, batch-to-batch variability, and a lack of quality control assays have curbed EV-based drug delivery and EV-derived therapeutic expansion in ophthalmology [49,115]. With the recent development of technology, more bioengineering strategies for EVs are explored within ocular contexts. For example, EV surface modification strategies are being developed to evade immunogenicity effects, overcome anatomical barriers, and refine targeting [68,72]. In addition, biomaterials as delivery platforms for EVs continue to be improved [95] along with other bio-based scaffolds [96]. Therefore, bioengineered EVs are promising therapeutic tools for ocular diseases. Future directions lie in the standardization of EV production and storage and in exploring better solutions for non-invasive drug delivery systems using bioengineered EVs.

## 7. Conclusions

EVs hold immense potential for treating eye disease, with their translation from bench to bedside currently constrained by challenges of vesicle heterogeneity, immunogenicity, and the anatomical barriers of the eye. The eye offers a uniquely compartmentalized and quantitatively accessible system, making ophthalmology an efficient testing platform for EV delivery, dosing and safety frameworks that can extend to neurological, inflammatory and systemic diseases. However, EV-based therapeutics for ophthalmologic applications require further development of standardized, scalable manufacturing processes and long-term clinical validation. In addition, EVs (natural and bioengineered) derived from non-ocular tissues need to be examined for native cargo composition to limit off-target effects. Improving drug delivery, batch-to-batch consistency, and tissue specificity, among the challenges noted in this review, highlights the significance of EV bioengineering. Good manufacturing practice (GMP) is critical in the development of pure, clinical-grade EVs for therapeutics. Bioengineered EVs from established cell lines hold promise in standardizing EV production due to superior reproducibility and scale-up over naturally derived EVs, enhancing the safety and quality of EVs for patient use. By employing engineering strategies such as surface modification, incorporation of biomaterials, or the development of synthetic and hybrid vesicles, EVs can be transformed from experimental platforms into precise therapeutic tools with broad translational potential.

## Figures and Tables

**Table 1:** Properties of MSCs, MSC-EVs, and iPSC-MSC-EVs.

	MSCs	iPSCs
Cells	Self-renewal [18] Multilineage differentiation [18] Immunomodulation capacity [18] Regenerative properties [18]	Self-renewal [28] Increased uniformity [28] Residual pluripotency [28]
EVs	Cell-free alternative [23,24] Increased biocompatibility [23] Lower immunogenicity [23,24] Variation in biological properties [24] Limited expandability [23,24]	Theoretically unlimited expandability [27] Comparable to parental MSCs [26] Prolonged effects vs. MSC-EVs [27]

(Abbreviations: MSCs, mesenchymal stem/stromal cells; EVs, extracellular vesicles; iPSCs, induced pluripotent stem cells.)

## Data Availability

Not applicable.
